# Isolated Dissection of the Ductus Arteriosus Associated with Sudden Unexpected Intrauterine Death

**DOI:** 10.3390/jcdd8080091

**Published:** 2021-07-31

**Authors:** Marny Fedrigo, Silvia Visentin, Paola Veronese, Ilaria Barison, Alessia Giarraputo, Erich Cosmi, Gaetano Thiene, Maria Teresa Gervasi, Cristina Basso, Annalisa Angelini

**Affiliations:** 1 Department of Cardiac, Thoracic, Vascular Sciences, and Public Health, University of Padua, 35128 Padua, Italy; marny.fedrigo@aopd.veneto.it (M.F.); ilaria.barison@unipd.it (I.B.); alessia.giarraputo@studenti.unipd.it (A.G.); gaetano.thiene@unipd.it (G.T.); cristina.basso@unipd.it (C.B.); 2 Department of Woman and Children Health, University of Padua, 35128 Padua, Italy; silvia.visentin.1@unipd.it (S.V.); paola.veronese@aopd.veneto.it (P.V.); erich.cosmi@unipd.it (E.C.); mariateresa.gervasi@aopd.veneto.it (M.T.G.)

**Keywords:** ductus arteriosus, remodeling, dissection of ductus arteriosus, sudden unexpected intrauterine death

## Abstract

We report five cases of sudden intrauterine death due to premature closure of the ductus arteriosus. In four cases, this was caused by dissecting the hematoma of the ductus arteriosus with intimal flap and obliteration of the lumen. In one case, the ductus arteriosus was aneurysmatic, with lumen occlusion caused by thrombus stratification. No drug therapy or free medication consumption were reported during pregnancy. The time of stillbirth ranged between 26 and 33 gestational weeks. We performed TUNEL analysis for apoptosis quantification. The dissecting features were intimal tears with flap formation in four of the cases, just above the origin of the ductus arteriosus from the pulmonary artery. The dissecting hematoma of the ductus arteriosus extended downward to the descending aorta and backward to the aortic arch with involvement of the left carotid and left subclavian arteries. TUNEL analysis showed a high number of apoptotic smooth muscle cells in the media in two cases. Abnormal ductal remodeling with absence of subintimal cushions, lacunar spaces rich in glycosaminoglycans (cystic medial necrosis), and smooth muscle cell apoptosis were the pathological substrates accounting for failure of remodeling process and dissection.

## 1. Introduction

Sudden intrauterine death is a socio-epidemiological problem. It accounts for 4–5/1000 births. The causes of unexpected death are mostly related to placenta injury in terms of placenta insufficiency, maternal malperfusion vascularization, inadequate fetal vascularization, or umbilical cord causes, such as nodal cord, rupture, marginal insertion, etc. [1,2]. Most of the studies in the literature show a high percentage of cases reported as unexplained causes, ranging from 20% to 50% [3].

Premature closure of the ductus arteriosus is a rare condition, which is difficult to diagnose. The incidence is not well defined [4,5,6,7,8]. Since 2013, our center has acted as a tertiary referral center in Northeast Italy for sudden unexpected intrauterine death. Among 375 cases collected since then, from 25 weeks of gestation to term, we identified five cases (1.3%) of abrupt ductus arteriosus closure caused by dissection of the ductus with hematoma of the parietal wall and obliteration of the lumen. These cases prompted us to review the literature on the topic of the ductus arteriosus and its remodeling process during fetal life to understand the structural abnormalities that could be at the base of these fatal events.

During fetal life, the ductus arteriosus, with its patency, guarantees blood flow from the right ventricle into the descending aorta bypassing the lungs since the role of blood oxygenation is played by the placenta.

The fetal ductus arteriosus is a large muscular artery originating from the 6th aortic arch [9,10,11], composed of three layers, the intima, the media with smooth muscle cells (SMCs), separated by elastic fibers, and the adventitia [12,13,14]. The intima is represented by a flat endothelium adherent to the internal elastic lamina or separated by intimal cushions. The intimal cushions are constituted by SMCs, usually longitudinally oriented with interposed thin elastic and collagen fibers and an extracellular matrix. By the end of gestation, they progressively become thicker. They are constantly present in all the ducts and preferentially located at the pulmonary extreme. The internal elastic lamina is a thick, continuous elastic layer, intact if adherent to the endothelium or fragmented, absent, or reduplicated when interposition of the intimal cushions occurs. The media are composed of many layers of SMCs, both contractile and synthetic, which are usually oriented circularly in the outer media but longitudinally in the inner media, favoring the postnatal contraction and shortening of the duct. Muscle cell layers are separated by fine reticulin fibers and thin elastic fibers. Mucopolysaccharides are interposed between the muscle and the elastic lamellae. Vasa vasorum are present in the outer layer of the media. During the second trimester of pregnancy, the media start to remodel, and the number of smooth muscle cells and extracellular matrix increases with gestational age. The ductal smooth muscle cells, longitudinally oriented, are inserted between the ending of the two great arteries’ elastic lamellae in the junctional zone. At this level, the elastic fibers of the pulmonary arteries interdigit with the SMCs of the ductal media. The ductal adventitia consists of fibroelastic connective tissue containing small blood vessels, nerves, and ganglia. The elastic fibers are most abundant adjacent to the media but do not form an external elastic lamina [13,15].

Two different conditions can affect the ductus arteriosus before birth: excessive patency with aneurysm formation and premature closure with lumen obstruction [16,17].

Aneurysm of the ductus arteriosus, a relatively rare lesion detected mostly in children and adults, can be incidental and asymptomatic but can evolve to spontaneous dissection, rupture, thrombosis, and embolism of the aorta and pulmonary arteries, leading to death [18,19,20,21,22,23,24,25]. Nowadays, thanks to routine diagnostic imaging modalities, these conditions can also be detected during fetal life.

Aneurysms of the ductus arteriosus can be congenital (present at birth) or acquired. Congenital ductus arteriosus aneurysms are reported to be more common than originally thought postnatal and can remain asymptomatic at birth [26,27,28].

Several theories have been postulated to explain the mechanism of congenital aneurysm formation of the ductus arteriosus [12,29,30]. The ductal aneurysm can resolve spontaneously without complications through the normal physiological constriction of the parietal wall of the duct at birth and or through a thrombus stratification inside the lumen with organization and incorporation of the thrombotic material and fibrotic occlusion [25]. However, during fetal life, the process of ductus arteriosus remodeling, which should allow postnatal closure, can fail and lead to aneurysm formation or persistent ductus arteriosus patency after birth [15,31,32,33].

The persistent ductus arteriosus has been associated with chromosomal abnormalities in 10% of the cases and TGFBeta signaling abnormalities, as in Marfan or Loeys Dietz, to TFAP2B in Char syndrome and to TFAP2B, TNF-RAF1, prostacyclin synthase in premature and term infants [31,34].

Few case reports present dissecting aneurysms of the ductus arteriosus as a cause of sudden unexpected intrauterine death.

The purpose of our study was to report the clinical and pathological characteristics of five cases of unexpected and sudden intrauterine death due to dissection of the ductus arteriosus and to elucidate the possible mechanism leading to this severe complication and ultimately to death.

## 2. Materials and Methods

Since 2013, our center at Padua University has acted as a tertiary referral center in the Veneto region for sudden intrauterine death. All the unexpected and sudden intrauterine deaths of the Veneto region are referred to Padua for autopsy, which are performed by two expert cardiovascular pathologists on rotation (AA, MF). The autopsy was a complete autopsy performed according to a standard adopted protocol for fetal autopsy inclusive of radiographic and photographic documentation. Clinical data from the mother and the pregnancy are reported in the clinical information protocol attached to the autopsy request. This protocol includes the metabolic and infective profile of the mother together with the family history and the documentation of the pregnancy. Routinely, the standard protocol for autopsy foresees histology of all the organs and if the maceration is not too advanced, we store fresh tissues for genetic studies when requested.

Among 375 cases collected since then, from 25 weeks of gestation to term, we have identified at autopsy, 5 cases (1.3%) of abrupt ductus arteriosus closure caused by dissection of the ductus with hematoma of the parietal wall and ab extrinseco compression of the lumen. We removed the thoracic block including the heart and the lungs and further evaluated them under a stereomicroscope. We used the sequential segmental approach for categorization. We opened the heart according to the blood flow. For histology, we sampled all the thoracic and abdominal organs. For the heart, we made a transverse cut to include the right ventricle, septum, and the left ventricle.

The ductus was sectioned with a serial transverse cut every 3 mm from the pulmonary junction to the aortic end, with a mean of three paraffin blocks per case. For each macroscopic block, we performed hematoxylin–eosin, elastic fiber–van Gieson, and Alcian–PAS staining. For apoptosis assessment, we performed TUNEL staining.

The protocol for sudden intrauterine death includes also the evaluation of the placenta according to the international guidelines “Sampling and definitions of placental lesions: Amsterdam placental workshop group consensus statement” [35].

## 3. Results

We present five cases of ductus arteriosus dissection, all male, as a cause of death (1.3%) from our registry of sudden intrauterine death since 2013. We used ReCoDe classification for causes of death [36] and according to this classification, we identified the 5 cases, which represent 7% of the causes in the fetus group (Group A). In Table 1, we present some information related to the mother. Unfortunately, we do not have any further information related to events following pregnancies.

Postmortem examination of the five cases.

At autopsy, the external examination showed a normally developed fetus for the gestational week (see Table 2). All the anthropometric indexes were in keeping with the gestational age. In four of the cases, the maceration of the fetus was severe.

**Figure 1:**
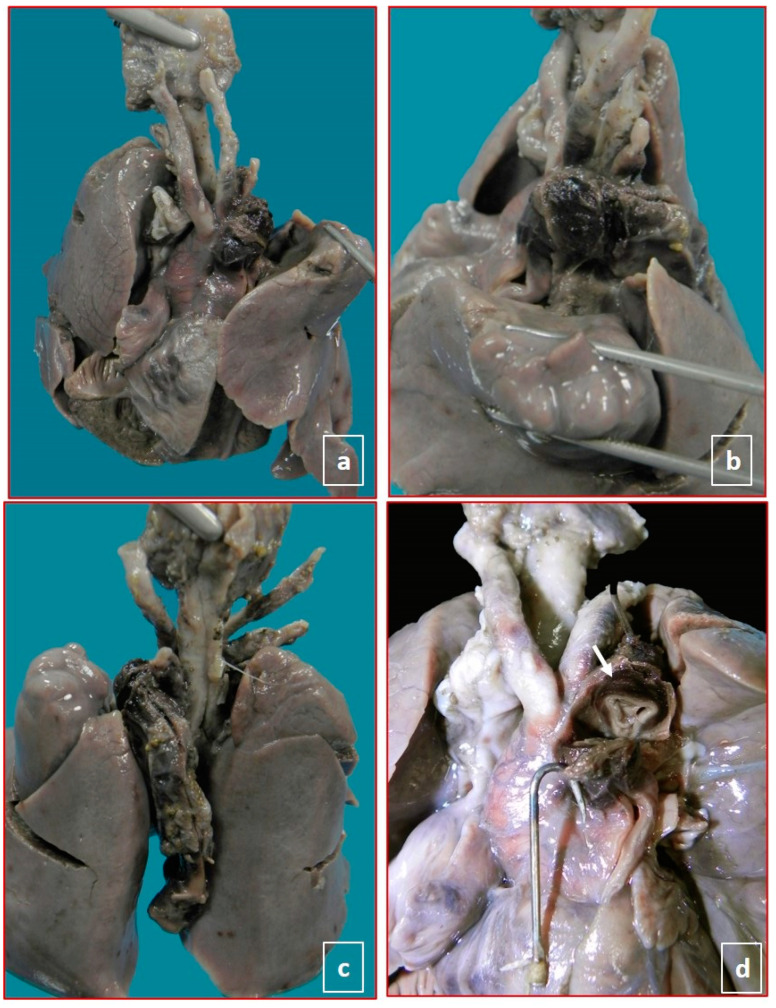
Male fetus, 32 gestation weeks (case 2), without congenital anomalies and slight intrauterine growth restriction. The placenta showed a fetal vascular malperfusion. No maternal pathology during this second pregnancy. (**a**–**c**), the localization and extension of peri-adventitial hematoma involving ductus arteriosus, aortic arch, brachiocephalic vessels, and descending aorta. (**d**), ab extrinseco compression of the aorta (white arrow).

**Figure 2:**
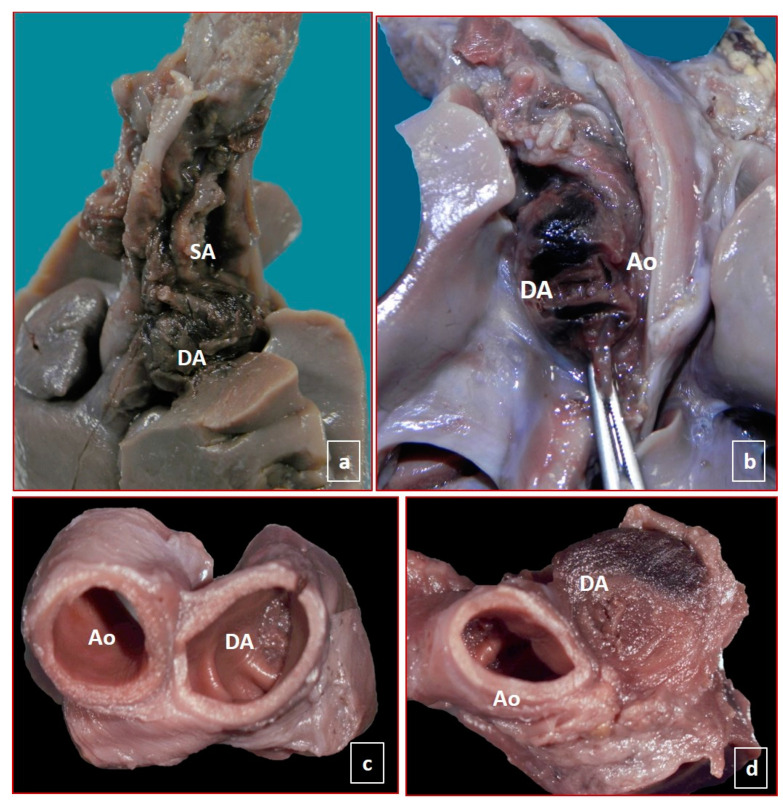
Male fetus, 33 gestation weeks (case 5), without congenital anomalies and slight intrauterine growth restriction. The placenta showed a high maternal vascular malperfusion. The mother had a positive history for hypertension and smoking during pregnancy. In (**a**,**b**), the localization and extension of peri-adventitia hematoma involved the ductus arteriosus, aortic arch, and brachiocephalic vessels. In (**c**,**d**), the complete closure of the ductus by thrombus. Ao: aorta; DA: ductus arteriosus.

Examination of the thorax and abdomen also showed normally related organs with a preserved normal laterality and no associated pathologies. No congenital defects were observed anywhere. In the thorax, after opening the pericardium, in four cases, adventitial hemorrhagic imbibition/hematoma at the level of the ductus arteriosus, giving an appearance of dilatation exceeding the diameter of the aorta and the pulmonary trunk, suggestive of dissection/hematoma was identified. Such hematoma started at the pulmonary end of the ductus arteriosus, partially involving the pulmonary trunk, the entire length of the ductus arteriosus, and extending into the descending aorta but stopped immediately after the junction with the ductus arteriosus. There was also the involvement of the origin of the left subclavian and left common carotid arteries (Figure 1 and Figure 2). The heart was located on the left side of the thorax, with the apex pointing to the left. The sequential segmental analysis showed situs solitus, concordant atrioventricular and ventriculoarterial connections, patent foramen ovale; only for one case (case 1), foramen ovale was restrictive, normally developed atrioventricular and semilunar valves. The coronary arteries were regular. No macroscopic abnormalities of the heart or great vessels were identified. The heart was opened according to the blood flow, and the pulmonary trunk showed a normal trifurcation, giving origin to the right and left pulmonary arteries and the ductus arteriosus. A serial transverse cut showed an intimal flap just above the junction of the ductus arteriosus with pulmonary vessels with a semi-circumferential involvement and dissection of the media-adventitia layer, which accounted for the adventitia hematoma detected from outside in four of the cases. In the other case, a ductal aneurysm was evident from the outside. A parietal hematoma completely occluded the lumen of the ductus arteriosus.

The lungs were normally located, the right trilobed, supplied by short, epi-arterial bronchus, and the left bilobed, supplied by a long, hypoarterial bronchus. The sectioning of the lung revealed, as expected, a compacted parenchyma, which, at histology, showed a saccular pattern in cases 1, 2, 3, and 5 and a canalicular pattern in case 4, in keeping with the gestational age. No vascular intraparenchymal abnormalities were present.

No emboli or infarcts were evident in the downstream circulation.

The placenta was available in four of the five cases. We could not detect any sign of infections; in two cases (case 4 and 5), we identified multifocal small infarcts. In another case (case 2), we recognized maternal vascular malperfusion. The last placenta (case 1) was unremarkable.

**Figure 3:**
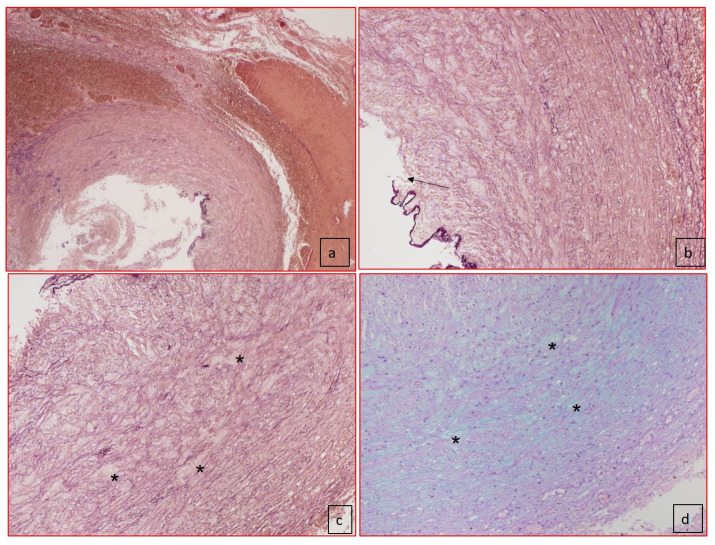
Histology of the ductus arteriosus (case 2). (**a**) Panoramic view of ductus arteriosus with elastic fiber–van Gieson staining showed the dissection of media (50× of magnification) and the absence of intimal cushions. (**b**) Elastic fiber–van Gieson staining at higher magnification showed the disorganization of elastic fibers and rupture of internal elastic lamina (black arrow). (**c**) Elastic fiber–van Gieson (100× magnification) staining showing disorganization of elastic fibers and presence of lacunar spaces, cystic medial necrosis (*), without SMCs replaced by mucopolysaccharides as shown in (**d**) with Alcian–PAS staining (100× magnification).

**Figure 4:**
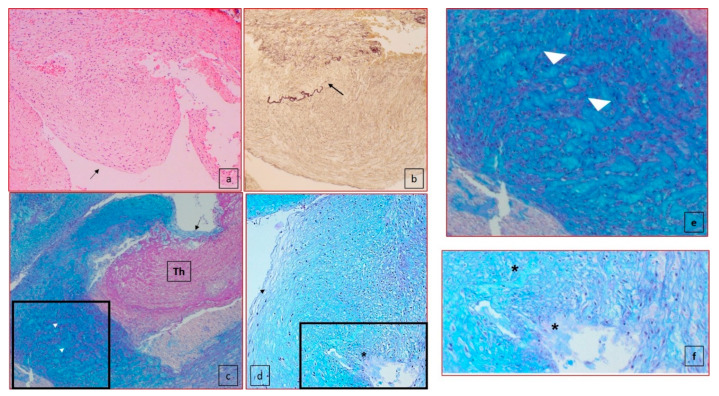
Histology of the ductus arteriosus. (**a**) Subendothelial intimal cushion (case 5) (50× magnification, black arrow); (**b**) elastic fiber–van Gieson (50× magnification) showed the interruption of the internal elastic lamina (black arrow); (**c**) presence of lacunar spaces (white arrow head) in the media closed to dissection (black arrow) (100× magnification) with thrombus formation (Th); (**d**) lacunar spaces, cystic medial necrosis (*) in the media, under the intimal proliferation (black arrow head) (100×). (**e**) High-power view of the insert highlighted in (**c**). (**f**) High-power view of the insert highlighted in (**d**).

**Figure 5:**
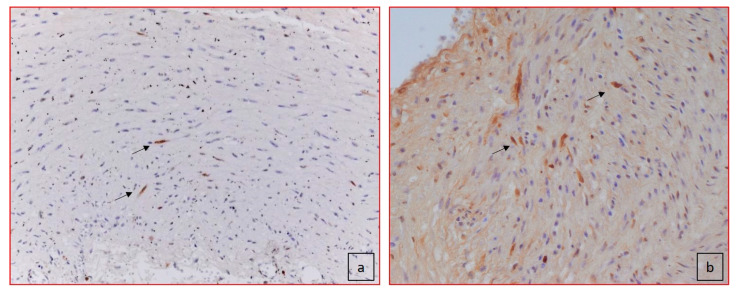
Apoptosis. Two different cases of TUNEL nuclear staining positivity for apoptosis of SMCs within the parietal wall (black arrows). (**a**) Case 2 (100×): apoptotic cells in the media without clear signs of proliferation; (**b**) case 4: diffuse apoptotic cells in the media (100×).

The microscopic examination of the ductus arteriosus showed absence of subendothelial intimal cushions with intact internal elastic lamina in three cases. In three cases, the presence of cystic medial necrosis (lacunar spaces in the media) were the prominent abnormalities (Table 3). Apoptosis in the media was present in two cases. In none of our cases, we identified inflammatory cell infiltration in the ductus arteriosus. In particular, each of our five cases presented a different association of the pathological substrates of the ductus arteriosus. In case 1, there was complete absence of intimal cushions, no media hematoma but aneurysm of the parietal ductal wall with thrombotic occlusion of the lumen, and no adventitia hematoma. In case 2, no subendothelial cushions, intimal flap with dissection of the outer media involving the adventitia, congestion of the vasa vasorum, large lacunar spaces, and apoptosis of SMCs in the media were identified (Figure 3). In case 3, we identified an intimal flap with thrombus stratification and lumen occlusion, and outer media dissection, adventitial hematoma and subendothelial cushion and medial large lacunar spaces with accumulation of glycosaminoglycans. In case 4, the youngest for gestational age, the ductus arteriosus was still without the remodeling of the subendothelial cushions as expected for the gestational age. Moreover, we identified an absence of lacunar space but a high rate of SMCs apoptosis in the media. An intimal flap with dissection of the outer media and hematoma of the adventitia was also present. In the fifth case, the substrate was characterized by large lacunar space in the presence of subendothelial cushions and intimal flap and parietal dissection and adventitial hematoma (Figure 4 and Figure 5).

## 4. Discussion

The results of our morphological study on five cases of stillbirth babies due to dissecting aneurysm of the ductus arteriosus showed that the dissection is similar to that affecting the aorta and the great vessel and other muscular arteries such as the coronary and cerebral arteries. We were able to identify an intima tear at the junction between the pulmonary trunk and the ductus with an intima-media flap and hematoma dissection through the media and the adventitia without any re-entry mechanisms in four of the five cases. This led to occlusion of the lumen by the flap segment and circumferential hematoma. In all the four cases, the hematoma dissection involved the descending aorta and the retrograde aortic arch. In only one case, we were unable to identify an intimal tear or parietal dissection, but the lesion was ascribed to ductal aneurysm and occlusive thrombus stratification.

There are no differences in terms of morphological substrates if we compare the dissection of the aorta or the coronary arteries with the dissection of the ductus arteriosus [37,38]. In both elastic and muscular arteries, an intimal tear/laceration can be identified with the blood entering the parietal wall and producing the intima-media or medial-adventitia detachment and creating a false lumen filled with blood, which determines the further progression of the dissection. The dissection can be either circumferential or semi-circumferential for the muscular arteries, while it is usually spiraliform for the aorta, according to the main longitudinal direction of the vessel and the orientation of the parietal wall SMCs of collateral vessels. Degenerative lesions such as mucoid pools, known as cystic medial necrosis, fragmentation of the elastic components and fragmentation and duplication of internal elastic lamina, and loss of SMCs are common features of the dissecting phenomena. In three of our cases, these lacunar spaces filled by extracellular mucoid matrix were the pathological substrates. Of relevance, in all of our cases, no inflammatory cell infiltration was present both at the site of intimal laceration and along the ductus, aorta, aortic arch, and the pulmonary trunk, excluding a possible vasculitis as a pathogenic mechanism of dissection. A thorough evaluation of the autopsy substrates as well as of the placenta also excluded the concomitant presence of other associated congenital anomalies, both cardiac and extracardiac, and confirmed the negativity of inflammatory status of the fetuses. Only in two of our cases, hypertension during pregnancy in the mother was reported and in one of them at preeclampsia range. We are aware that hypertension in the mother or preeclampsia status could affect the placenta causing vascular malperfusion and fetal growth restriction, with the evidence of parietal wall stiffness as an indirect sign of intravascular stress [39]. However, we do not know if this mechanism could have played a role in our cases. In the history of the mother, we did not find other risk factors traditionally recognized for dissection in adults. We cannot exclude a genetic background accounting for connective tissue disorders, dissection, and aneurysm formation since we could not perform genetic studies on these cases, mainly in relation to the severe maceration in four of them. We plan to carry out a genetic assessment for the most frequent gene involved in collagen disease in the case with more preserved conditions [31,40].

During pregnancy, the ductus arteriosus remains open, and during the second trimester, the proliferation of intimal cushions with lumen reduction will prepare for ductus arteriosus closure. The lumen is stellate in the closing duct and is reduced in size, mainly in the pulmonary extremity [9,10,11,12,13,15,24,25].

Remodeling for the preparation of ductus arteriosus closure at birth with immediate contraction of the parietal wall implies differentiation of vascular SMCs and endothelial cells, production of the extracellular matrix, vascular SMCs proliferation and migration, and finally, a decrease in elastic fibers and an increase in fibrous connective tissue [12,25]. The sequence of events can be summarized as follows: endothelial detachment from the internal elastic lamina with an accumulation of extracellular matrix in the subendothelial space, migration of SMCs from the media into the intima through the fenestration of the elastic lamina, growth of endothelial cushions, occlusion of the lumen by contraction and overgrowth of cushions and the necrosis of the inner layer of the media, and degeneration of the ductus arteriosus into a fibrous ligament at birth through apoptosis and necrosis [9,13,15,24,25]. In preterm infants, the ductus arteriosus usually remains open because there is a lack of intimal cushions. In the case of persistent ductus arteriosus or patent ductus arteriosus in a full-term fetus, the intimal cushions are not so well formed. They are encircled by an additional subendothelial elastic lamina.

In the congenital aortic arch aneurysm, the ductus arteriosus is usually tortuous, saccular, and dilated and elongated, and characterized microscopically by a lack of intimal cushion formation and defective elastic lamellae formation, enhancement of necrosis, and mucoid degeneration of the inner part of the media [12,15]. This remodeling process occurs in the third trimester of pregnancy. They are often associated with connective tissue disorders such as Marfan syndrome or Ehlers Danlos syndrome [34]. They can resolve or evolve with fatal complications such as thromboembolic extension into the main pulmonary artery or the aorta, spontaneous rupture, erosion or compression of adjacent structures, and infection [24,25,41,42,43].

In all the other 370 cases of our registry, we did not identify the presence of aneurysms of the ductus arteriosus. Dissecting aneurysm of ductus arteriosus can be regarded as the end of the spectrum of the patent ductus arteriosus (PDA), a congenital cardiac abnormality that can be a single cardiac abnormality or can be part of other associated congenital heart diseases. PDA is characterized by the lack of closure at the time of birth with the persistence of patency, allowing shunting of blood between the systemic and the pulmonary circulations [31].

At a histomorphological analysis, the patent ductus arteriosus is characterized by the absence of the features at the base of remodeling for closure after birth, which are the intimal cushions, pads of proliferating SMCs migrating from the media into the intima, and thickening of the medial wall as a consequence of longitudinal SMCs fascicles arrangement in the subintimal space [15,30,31]. The absence of intimal cushions in three of our dissecting cases supports the theory of abnormal remodeling for postnatal ductus arteriosus closure, with the weakening of the parietal wall, which can favor secondary dissection. If the dissection were not occurring, the result would be eventual patency of the ductus arteriosus. Moreover, the presence of large lacunar spaces rich in glycosaminoglycans is recognized as the cystic medial necrosis substrate in aortic dissection.

Dissecting aneurysm is an acute event that has led to fetal death. Since this was an unexpected and sudden event, we do not know the exact time frame for the dissection of the parietal wall, occlusion of the ductus arteriosus, and death. The barrage at the ductus arteriosus would have caused a redirection of flow toward the pulmonary arteries and an increase in blood shunt from the right atrium to the left atrium. The dimension of the foramen ovale would play a crucial role in the hemodynamics of these fetuses. Very few papers have reported dissection of the ductus arteriosus as the cause of sudden unexpected deaths in single-center experiences or as anecdotal cases without an accurate and detailed description of the parietal dissected wall [21,41,42].

One point should be discussed as well in relation to the terminology adopted in the literature. As in adults with aortic dissection, in which we are not utilizing anymore the term dissecting aneurysm, since the dissection has been recognized to occur in the absence of ectasia or aneurysm formation, it would be more appropriate to adopt the term dissection of ductus arteriosus rather than dissecting aneurysm. The dilatation is not a prerequisite for dissection, but dissection can manifest without an increase in the diameter of the vessel.

On the other hand, an aneurysm of the ductus arteriosus can be present and be detected accidentally during the fetal ultrasound and resolved spontaneously after birth with closure without complications [44].

Several theories have been proposed for the development of an aneurysm of the ductus arteriosus. At the time of birth, delayed closure of the aortic end of the ductus would expose the ductal wall to high pressure with related dilatation. During the third trimester, as detected by ultrasound, dilatation and aneurysm development has been ascribed to the weakening of the parietal wall with a reduction of intimal cushion formation or deposition of elastin. Aneurysm development in some reported cases was associated with defective fibrillin or collage [34,41] or vascular SMCs dysregulation [31,34]. In our cases, we could not carry out a genetic study to unravel the possible genetic defects associated with dissection. However, no associated cardiac or extracardiac anomalies during the autopsy were present in our cases. Aneurysms of the non-patent ductus arteriosus have been reported by Falcone et al. to affect patients at different ages but all after birth from infants to adults and related to death in 31% of the reported cases [20].

To the best of our knowledge, a ductal aneurysm has never been described in the stillborn fetus.

Spontaneous premature closure of the fetal ductus can be a cause of fetal demise. However, in the cases reported in the literature, no morphological description of the mechanism of closure has been described [4,16,45,46]. Much attention in those cases was driven toward the hemodynamic consequence of the closure, with an ultrasound description at the ductus level, with right heart failure, pulmonary overflow, and persistent pulmonary hypertension after birth in case of successful delivery. The mechanism of premature closure is still ill-defined. We cannot exclude that in some of these cases, a dissecting aneurysm could be responsible per se for the lumen occlusion.

Some other aspects are puzzling in our five cases. All of them were males. However, we do not know if this is by chance or it implies any male gender propensity to dissection. All of them except one occurred at around 32 gestational weeks, which is the time of the remodeling process of the arterial duct, with intimal cushion formation in preparation for physiological closure occurring at birth. This could imply failure of the remodeling process.

## 5. Study Limitation

The main limitation is represented by the poor quality of the autoptic material on which we performed the histological and genetic assessments, in part due to the severe maceration of four of the five cases. As a consequence of the high level of degradation of DNA, it was not possible to detect possible genetic collagenopathies. Indeed, these genes are characterized by long nucleotide sequences.

## 6. Conclusions

In case of sudden unexpected intrauterine death, a thorough autopsy should be performed to identify rare conditions such as dissecting ductus arteriosus as the cause of death. Histological examination should be performed for the detection of pathological substrates accounting for intimal tears, dissection, hematoma, apoptosis, and absence of inflammatory infiltrate. Further genetic studies should be carried out to confirm or exclude the genetic background.

## Figures and Tables

**Table 1:** Clinical data.

Case	Sex	GA	Ethnicity	Risk Factors of Mother	Pregnancy	Medications during Pregnancy	Reported Symptoms during Pregnancy
1	M	33	Caucasian	Smoking during pregnancy	First	None	None
2	M	32	Caucasian	No risk factors	Second	None	None
3	M	32	Caucasian	Hypertension, smoking during pregnancy	First	None	Hypertension
4	M	26	Caucasian	No risk factors	First	None	None
5	M	33	Caucasian	Pre-eclampsia	First	None	Hypertension

GA: gestational age.

**Table 2:** Autopsy major findings.

Case	Body Weight	Heart Weight	Wall Thickness RV/S/LV mm	GM	DA Anatomical Macroscopic Description	Foramen Ovale
1	1750 g	8.7 g	2.5/2.8/2.8	III	Dissecting DA	Restrictive
2	1650 g	8.5 g	2.4/2.5/2.5	III	Dissecting DA	Patent
3	1300 g	7 g	2/2/2.2	III	Dissecting DA	Patent
4	810 g	5 g	1.2/1.3/1.8	III	Dissecting DA	Patent
5	1670 g	9 g	2.3/2.5/2.5	I	Dissecting DA	Patent

RV: right ventricle; S: septum; LV: left ventricle; GM: grade of maceration; DA: ductus arteriosus.

**Table 3:** Microscopic features of the ductus arteriosus.

Case	Intimal Flap	Intima	Media-Adventitia	Inflammation	Apoptosis (TUNEL)
1	Absent	No subendothelial cushions, thrombotic lumen occlusion	Outer dissecting hematoma, no cystic medial necrosis	Absent	+
2	Present	No subendothelial cushions	Outer dissecting hematoma, cystic medial necrosis	Absent	+++
3	Present	Subendothelial cushions, thrombus stratification	Outer dissecting hematoma, cystic medial necrosis	Absent	+
4	Present	No subendothelial cushions	Outer dissecting hematoma, no cystic medial necrosis	Absent	+++
5	Present	Subendothelial cushions	Dissecting hematoma, cystic medial necrosis	Absent	+

## Data Availability

Not applicable.
